# Characterization of the complete chloroplast genome of *Pellionia scabra* (Urticaceae)

**DOI:** 10.1080/23802359.2022.2124823

**Published:** 2022-09-27

**Authors:** Xuelian Yang, Li Yan, Xia Wang, Yongfei Wu, Xiaojing Hu

**Affiliations:** College of Agriculture, Guizhou University, Guiyang, China

**Keywords:** *Pellionia scabra*, complete chloroplast genome, phylogenic analysis, Illumina, Urticaceae

## Abstract

*Pellionia scabra* Benth. 1861 (Urticaceae) is distributed in east and southeast Asian countries, including China, Vietnam, and Japan, and has important applications in construction, medicine, and the food industry. We sequenced the genome using Illumina DNA sequencing technology. The genome was 153,220 bp long. Annotation of the genome showed that it encoded 130 genes, including 85 protein-coding genes, 37 tRNA genes, and eight rRNA genes. In addition, 15 of the genes contained a single intron and two contained two introns. Furthermore, *rps*12 consists of 3 exons that are expected to be trans-spliced together. Subsequent phylogenetic analysis revealed that *P. scabra* is closely related to the *Elatostema* species (i.e. *E. stewardii* [MZ292972], *E. dissectum* [MK227819], and *E. laevissimum* var. *laevissimum* [MN189961]).

*Pellionia scabra* Benth. 1861 (Urticaceae) is distributed in east and southeast Asian countries, including China, Vietnam, and Japan. The species is popular for its use in interior decoration and as a source of traditional Chinese medicine. In addition, *P. scabra* is a popular wild vegetable (Wang et al. 2013). The aim of the present study was to improve the current understanding of the species’ characteristics and functions by sequencing its complete chloroplast (cp) genome and by performing a phylogenetic analysis of 77 common protein-coding genes from the cp genomes of *P. scabra* and 28 other species.

Leaves of *P. scabra* were collected from Guizhou Botanical Garden (N 26°37′20″, E 106°43′29″) and stored samples (accession no. MCC20210902YX) from the Laboratory of the College of Agriculture, Guizhou University (Guiyang, China; contact person: Xuelian Yang, email: yxl1299927812@outlook.com). The study was approved by Guizhou University and performed in accordance with the national Wild Plant Protective Regulations.

Total genomic DNA was extracted from 150 mg fresh leaves using the cetyltrimethylammonium bromide method (Doyle and Doyle 1987). An aliquot of purified DNA (0.5 μg) was then fragmented to construct a short-insert (350 bp) library using the Nextera XT DNA library preparation kit (Illumina, San Diego, CA). The library was sequenced using the Illumina NovaSeq 6000 platform, and the raw data were edited using the NGS QC Tool Kit v2.3.3 (Patel and Jain 2012). High-quality sequence data (3912 Mb) were then selected for the *de novo* assembly of the complete cp genome using the assembler SPAdes v3.11.0 (Bankevich et al. 2012). Finally, the complete cp genome was annotated using PGA (Qu et al. 2019) with the cp genome of *Elatostema stewardii* as a reference.

The complete cp genome of *P. scabra* (153,220 bp) exhibited a typical quadripartite structure, including a large single-copy (LSC) region (84,480 bp), small single-copy (SSC) region (17,568 bp), and pair of inverted repeat (IR) regions (IRa and IRb, each 25,586 bp). The cp genome’s total GC content was 36.4%, and the genome contained 130 genes, including 85 protein-coding genes, 37 tRNA genes, and eight rRNA genes. In addition, 15 of the genes (*trn*K-UUU, *rps*16, *trn*G-UCC, *atp*F, *rpo*C1, *trn*L-UAA, *trn*V-UAC, *pet*B, *pet*D, *rpl*16, *rpl*2, *ndh*B, *trn*I-GAU, *trn*A-UGC, and *ndh*A) contained a single intron and two (*ycf*3 and *clp*P) contained two introns. Furthermore, *rps*12 consists of three exons that are expected to be trans-spliced together.

To analyze the *P. scabra* cp genome, the complete cp genomes of 28 other plant species, including 27 other members (16 genera) of Urticaceae and one outgroup taxon (*Citrus aurantifolia*, Rutaceae; KJ865401), were obtained from NCBI. The sequences of 77 common protein-coding genes from *P. scabra* cp genome and the other 28 other complete cp genomes were aligned using MAFFT version 7.037 (Katoh and Standley 2013) with the FFT-NS-2 strategy. A phylogenetic tree of the 29 cp genomes was then constructed using RAxML version 8.2.9 (Stamatakis 2014), based on the maximum-likelihood method with 1000 bootstrap replications and the TVM + F+I + G4 model, which was selected using ModelFinder version 1.6 (Kalyaanamoorthy et al. 2017).

Phylogenetic analysis indicated that *P. scabra* is closely related to the *Elatostema* species (i.e. *E. stewardii* [MZ292972], *E. dissectum* [MK227819], and *E. laevissimum* var. *laevissimum* [MN189961]; Figure 1). In addition, *Pellionia* and *Elatostema* were more closely related to the genera *Procris*, *Pilea*, and *Urtica* than to the other genera, and the genera *Oreocnide*, *Rousselia*, *Hemistylus*, *Pouzolzia*, *Pipturus*, *Boehmeria*, *Debregeasia*, and *Droguetia* formed a distinct clade.

**Figure 1.**
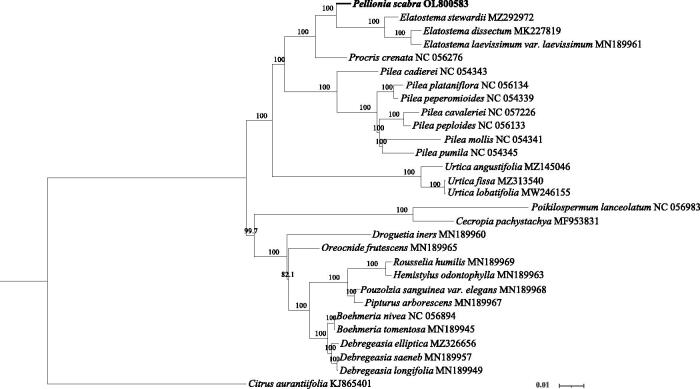
Maximum-likelihood tree of protein-coding gene sequences from the complete chloroplast sequences of *Pellionia scabra* Benth.1861 (Urticaceae) and 28 other plant species. The analysis was performed using 77 homologous protein-coding genes. Node values indicate bootstrap support (1000 replicates).

The results of this study provide information about the complete cp genome of *P. scabra*, thereby facilitating further research on the species’ clinical and commercial applicability.

## Data Availability

The genome sequence data that support the findings of this study are openly available in GenBank (https://www.ncbi.nlm.nih.gov/) under accession number OL800583. The associated BioProject, SRA, and Bio-Sample numbers are PRJNA787431, SRR17177970, and SAMN23802974, respectively.
